# Comparative Study on Burden, Features and Determinants of Disorders of Gut‐Brain Interaction Between Southern Europe and the Rest of Continent: Results From the Rome Foundation Global Epidemiology Study

**DOI:** 10.1002/ueg2.70226

**Published:** 2026-05-05

**Authors:** Giovanni Marasco, Keren Hod, Luigi Colecchia, Ami D. Sperber, Olafur S. Palsson, Shrikant I. Bangdiwala, Giovanni Barbara

**Affiliations:** ^1^ Department of Medical and Surgical Sciences University of Bologna Bologna Italy; ^2^ IRCCS Azienda Ospedaliero‐Universitaria di Bologna Bologna Italy; ^3^ Department of Nutritional Sciences School of Health Sciences Ariel University Ariel Israel; ^4^ Assuta Medical Centers Tel Aviv Israel; ^5^ Faculty of Health Sciences Ben‐Gurion University of the Negev Beer‐Sheva Israel; ^6^ Center for Functional GI & Motility Disorders Chapel Hill North Carolina USA; ^7^ Department of Health Research Methods Evidence and Impact McMaster University Hamilton Ontario Canada; ^8^ Population Health Research Institute McMaster University Hamilton Ontario Canada

**Keywords:** disorders of gut‐brain interaction, epidemiology, Europe, functional constipation, functional diarrhea, functional dyspepsia, irritable bowel syndrome, Mediterranean diet

## Abstract

**Background and Aim:**

Disorders of gut–brain interaction (DGBI) are highly prevalent worldwide. Although the epidemiology of DGBIs in Europe has been previously investigated, data comparing disease prevalence across European regions in relation to sociodemographic and lifestyle factors are lacking. Therefore, this study aimed to assess the prevalence, regional distribution, and associated factors of DGBI in Southern Europe, and to compare findings with other European regions.

**Methods:**

Data were drawn from the Rome Foundation Global Epidemiology Study (RFGES). A representative sample of 20,420 European adults completed a comprehensive internet‐based questionnaire assessing DGBI presence, psychological distress, somatic symptoms, dietary habits, and healthcare utilization. Comparative analyses were conducted between Southern European countries and the rest of the continent (Northern, Western, and Eastern European countries). Multivariate logistic regression identified independent associated factors.

**Results:**

The prevalence of adults with at least one DGBI was significantly higher in Southern Europe than in the rest of Europe (44.0% [42.4–45.5] vs. 39.0% [38.3–39.8]; *p* < 0.001). Irritable bowel syndrome and functional dyspepsia were more prevalent in Southern Europe than in the rest of Europe. Similar trends were found for functional constipation and functional diarrhea. Individuals with DGBI in Southern Europe showed higher psychological distress but lower somatic symptom burden and lower work productivity and activity impairment. In multivariable models, residence in Southern Europe, female sex, younger age, higher psychological distress, greater somatic symptom burden, higher educational attainment, urban residence and more frequent healthcare utilization were independently associated with DGBI.

**Conclusions:**

Southern European populations exhibit a higher prevalence of DGBI compared with other European regions. Individuals with DGBI in Southern Europe showed higher rates of psychological distress, whereas those in the rest of Europe showed higher somatic symptom burden, greater work productivity and activity impairment. DGBI rates in Europe appear to be driven by a multifactorial interplay of demographic and psychosocial factors.

## Key summary

- What is known
  ◦ Disorders of gut–brain interaction (DGBI) are highly prevalent worldwide, affecting approximately 40% of the adult population.
  ◦ DGBIs are multifactorial conditions involving altered gut motility, visceral hypersensitivity, microbiota changes, immune activation, and dysregulation of the gut–brain axis.
  ◦ Psychological distress and somatic symptom burden are strongly associated with the presence and severity of DGBI.
  ◦ Regional and lifestyle‐related factors may influence the epidemiology of DGBI, although European comparative are scarce.
- What this study adds
  ◦ Southern European countries (Italy and Spain) show a significantly higher prevalence of at least one DGBI compared with Northern, Western, and Eastern European regions.
  ◦ Specific conditions, including irritable bowel syndrome, functional dyspepsia, functional constipation, and anorectal DGBIs, are more frequent in Southern European populations.
  ◦ Higher psychological distress and greater healthcare‐seeking behavior in Southern Europe may contribute to the increased reporting and burden of DGBIs.
  ◦ Factors associated with DGBIs across Europe include female sex, younger age, urban residence, psychological distress, higher educational attainment, more frequent healthcare utilization and increased somatic symptom burden, reinforcing the biopsychosocial framework of these disorders.

## Graphical abstract

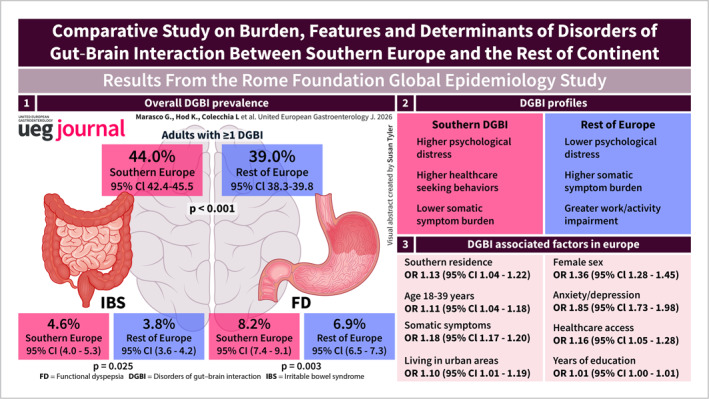

## 1 Introduction

Disorders of gut–brain interaction (DGBI) encompass a broad range of chronic gastrointestinal conditions characterized by symptom clusters without identifiable structural abnormalities [1, 2, 3]. These conditions result from a combination of motility disturbances, visceral hypersensitivity, changes in mucosal and immunological function, altered gut microbiota, altered central nervous system processing of afferent sensory input and genetic polymorphism [4, 5, 6, 7]. Irritable bowel syndrome (IBS), functional dyspepsia (FD), functional constipation (FC) and functional diarrhea (FDr) are the most common conditions that fall within the DGBI spectrum [4, 8]. Although the burden of individual DGBI worldwide has been quantified, no study to date has provided a thorough assessment of DGBI burden in Europe, specifically comparing Southern European regions with the rest of the continent. The Rome Foundation Global Epidemiology Study (RFGES) evaluating a total of 22 DGBIs revealed that 40.3% of 54,127 participants in 26 countries met diagnostic criteria for at least one DGBI, implying the major global burden of these disorders [9]. The RFGES study in Europe included respondents, of whom about 40% had at least one DGBI [9]. However, these data do not take into account regional variability within the continent. In fact, Southern European countries differ markedly from the rest of the continent in terms of healthcare systems, socioeconomic conditions, cultural profiles, and lifestyle patterns. Psychosocial determinants are particularly relevant, as Southern European populations have experienced greater exposure to economic instability and unemployment over recent decades, variables associated with increased psychological distress and altered gut–brain axis function [10, 11]. These elements may amplify symptom perception and chronicity, thereby increasing the measurable burden of DGBIs. Cultural dimensions further modulate this landscape. In Southern Europe, symptom expression tends to be more somatically oriented, and gastrointestinal complaints are often more readily externalized, influencing both reporting patterns and healthcare‐seeking behavior, potentially leading to higher DGBI rates. On the other hand, Southern European countries are worldwide renowned for the Mediterranean lifestyle and diet, which has been associated with the general subject well‐being, reduced cardiovascular and metabolic disorders and reduced rates of psychological disorders, the latter being associated with higher prevalence rates of DGBI [12, 13]. The Mediterranean diet, characterised by a high intake of fruits, vegetables, nuts, olive oil, whole grains and moderate fish, with a low consumption of red and processed meats, is associated with beneficial shifts in the gut microbiota and reductions in intestinal inflammation [14, 15, 16]. In individuals with gastrointestinal disorders (including IBS and FD), greater adherence to the Mediterranean diet has been linked to lower prevalence or severity of symptoms, suggesting that its composition and anti‐inflammatory gut‐modulating effects may ameliorate gastrointestinal symptoms [17].

In light of this contrasting background, we aimed to report the burden of DGBI in Southern Europe, comparing it with the rest of the continent. In addition, we aimed to describe similarities and differences between patients with a DGBI diagnosis in Southern Europe and the rest of Europe. Finally, we aimed to assess DGBI risk factors in Europe, focusing on sociodemographic, psychological, dietary and other lifestyle factors.

## 2 Methods

### 2.1 Study Design and Participants

This study is based on data from the RFGES, a large multinational cross‐sectional survey aimed at assessing the prevalence and burden of disorders of DGBI. The complete methodology of the RFGES has been previously published and described in detail elsewhere [9]. In brief, the study collected data from 73,076 adult participants (≥ 18 years) across 33 countries via either internet‐based or face‐to‐face interviews. For the current analysis, only data obtained through internet‐based self‐administered questionnaires were used (*n* = 54,127). Participants were drawn from Qualtrics Ltd. (Provo, Utah), a pre‐existing online panel of individuals who had consented to participate in health‐related research. To minimize selection bias, the survey was introduced as a general health study without any mention of gastrointestinal or psychological symptoms. All participants provided electronic informed consent prior to participation, and data were collected anonymously. The study received ethics approval or was deemed exempt in each participating country.

**FIGURE 1:**
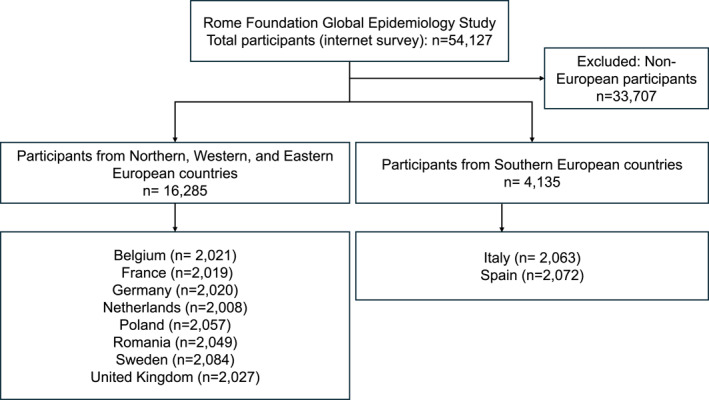
Flow chart showing the selection of participants for the analysis from the Rome Foundation Global Epidemiology Study (*N* = 54,127). Participants from countries outside of Europe (*n* = 33,707) were excluded. The remaining participants (*n* = 20,420) were grouped into: (1) Northern (Sweden, United Kingdom), Western (Belgium, France, Germany, Netherlands), and Eastern European countries (Poland, Romania); and (2) Southern European countries (Italy and Spain).

In Europe, a representative sample of 20,420 adults was recruited. The European sample was further categorized into Southern European countries (Spain, Italy), and with respondents from Northern (Sweden, United Kingdom), Western (Belgium, France, Germany, Netherlands), and Eastern European countries (Poland, Romania), as classified according to FAO regional groupings [18]. Data from Northern, Western and Eastern European countries were subsequently pooled and analyzed as a single dataset (Figure 1).

## 3 Data Collection and Variables

DGBI was defined as the presence of at least one DGBI, diagnosed using validated modules from the Rome IV Diagnostic Questionnaire [19] (alongside a self‐reported checklist to rule out organic diseases and surgeries that could account for gastrointestinal symptoms). DGBI were categorized by anatomical regions (esophageal, gastroduodenal, bowel, anorectal). Specific DGBI diagnoses, including IBS and its subtypes [IBS with predominant constipation (IBS‐C), IBS with predominant diarrhea (IBS‐D), mixed IBS (IBS‐M) and undefined IBS (IBS‐U)], FD, FC, and FDr, were assessed individually. A detailed description of the administered questionnaires and classifications used in text are available in the Supporting Information S1. In brief, somatic symptoms were evaluated using the Patients Health Questionnaire‐12 (PHQ‐12) [20], physical and mental health‐related quality of life was assessed via the Patient‐Reported Outcomes Measurement Information System (PROMIS) Global‐10 [21], the work productivity and activity Impairment was assessed via the General Health (WPAI:GH) questionnaire [22], psychological distress was evaluated using the Patients Health Questionnaire‐4 (PHQ‐4) [23], and the weekly consumption frequency of various food groups was assessed via the Food Frequency Questionnaire (FFQ).

### 3.1 Statistical Analyses

All eligible participants from internet interviews were included. Data normality was evaluated using the Kolmogorov‐Smirnov test; normality assessments were supplemented by visual inspection of histograms and Q‐Q plots. Normally distributed continuous variables were reported as mean and 95% confidence interval (CI), otherwise, as median, and 95% CI. Categorical variables were reported as percentages and 95% CI. Age categories were categorized as 18–39, 40–64, and 65+ years. The prevalence rate of each one of the 22 DGBIs was calculated for the total European population and was stratified by the two European macro‐regions. Differences between Southern versus Northern, Western and Eastern European countries in terms of demographics, clinical features and lifestyle factors were calculated using Student's T test, Mann‐Whitney, Fischer test, and Chi‐square as appropriate. To further evaluate regional heterogeneity within Europe, analyses were extended to compare Northern, Western, Eastern, and Southern European regions separately. For each of the 22 DGBI diagnoses, overall differences were assessed using chi‐square tests. When statistically significant, pairwise comparisons between regions were performed using adjustment for multiple testing using the Holm method.

Multiple multivariable logistic regression models using the forward stepwise selection method were applied to clarify the role of sociodemographics, clinical and psychological features, dietary factors, healthcare access, and region of residence in relation to the occurrence of at least one DGBI or FD or IBS in Europe. The estimated odds ratios (OR) with their 95% CI were calculated. Independent variables that were inserted into these models were those with a significant association with DGBI (*p* < 0.1), as found by a previous univariate analysis. Independent variables that were highly correlated (*r* ≥ 0.600) were not included in these multivariable logistic regression models simultaneously, even if they were significantly associated with DGBI, to avoid multicollinearity. Associations with a *p*‐value < 0.05 were considered statistically significant. The statistical analysis was performed using SPSS Statistics for Windows package, version 30.0 (IBM Corp. Released 2019, Armonk, NY).

## 4 Results

### 4.1 Sociodemographic Characteristics Associated With DGBI

**FIGURE 2:**
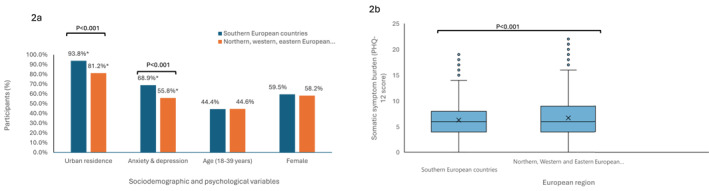
Sociodemographic, psychological characteristics, and somatic symptom burden in individuals with at least one DGBI across European regions. (2a) Comparison of selected sociodemographic and psychological variables between participants with at least one DGBI from Southern European countries and those from Northern, Western, and Eastern European countries. Data are expressed as percentages of participants. Significant regional differences were observed for urban residence (93.8% vs. 81.2%, *p* < 0.001) and presence of anxiety and depression (68.9% vs. 55.8%, *p* < 0.001). No significant differences were found for age Group 18–39 years (44.4% vs. 44.6%) or female sex (59.5% vs. 58.2%). (2b) Distribution of somatic symptom burden in individuals with at least one DGBI measured by the PHQ‐12 score across European regions. Box plots represent median and interquartile ranges, with whiskers indicating variability and dots representing outliers; the cross denotes the mean value. Participants from Southern European countries exhibited a significantly lower somatic symptom burden compared with those from Northern, Western, and Eastern European countries (*p* < 0.001).

Overall, the population with DGBIs was predominantly female (58.5%), with a mean age of 43.8 years. No significant differences were observed between Southern and other European regions with respect to sex distribution, mean age, or age‐group stratification. In contrast, marked regional differences emerged for education and body mass index (BMI). Participants with at least one DGBI from Southern Europe had significantly higher educational attainment (mean 14.9 vs. 12.8 years, *p* < 0.001) and lower BMI (25.1 vs. 26.1 kg/m^2^, *p* < 0.001) compared with their Northern, Western, and Eastern European counterparts. Notably, obesity was significantly more frequent in the other European regions (20.8% vs. 13.7%, *p* < 0.001). Urban residence was substantially more common in Southern Europe (93.8% vs. 81.2%, *p* < 0.001), with a higher proportion living in large cities and a markedly lower representation of village or rural residents (Table 1; Figure 2).

**TABLE 1:** Sociodemographic data of the European population with at least one DGBI.

	Overall European population with at least one DGBI (*n* = 8170)	Southern European countries^a^ with at least one DGBI (*n* = 1818)	Northern, Western and Eastern European countries^b^ with at least one DGBI (*n* = 6352)	*p* value
Female, % (95% CI)	58.5 (57.4–59.6)	59.5 (57.2–61.8)	58.2 (57–59.4)	0.335
Age (years), mean (95% CI)	43.8 (43.5–44.1)	43.7 (43–44.4)	43.8 (43.4–44.2)	0.788
Age groups, % (95% CI)				
18–39 years	44.6 (43.5–45.7)	44.4 (42.1–46.7)	44.6 (43.4–45.9)	0.866
40–64 years	41.5 (40.4–42.6)	40.6 (38.3–42.9)	41.8 (40.5–43)	0.392
65+ years	13.9 (13.2–14.7)	15 (13.4–16.8)	13.6 (12.8–14.5)	0.134
Education (years), mean (95% CI)	13.3 (13.2–13.4)	14.9 (14.7–15.2)	12.8 (12.7–13.0)	< 0.001
BMI (kg/m^b^),mean (95% CI)	25.9 (25.7–26)	25.1 (24.9–25.4)	26.1 (25.9–26.2)	< 0.001
BMI levels^c^, % (95% CI)				
Underweight	4.5 (4.0–5.0)	4.6 (3.7–5.8)	4.4 (3.9–5.0)	0.768
Normal weight	45.9 (44.8–47.0)	50.6 (48.2–53.0)	44.5 (43.2–45.8)	< 0.001
Overweight	30.5 (29.5–31.6)	31.1 (28.9–33.4)	30.3 (29.1–31.5)	0.552
Obesity	19.1 (18.2–20.0)	13.7 (12.1–15.4)	20.8 (19.7–21.9)	< 0.001
Current family status, % (95% CI)				
Single	26.8 (25.8–27.8)	25.6 (23.6–27.7)	27.1 (26–28.2)	0.199
Married	44.8 (43.8–45.9)	50.6 (48.2–52.9)	43.2 (42–44.4)	< 0.001
Divorced	8.1 (7.5–8.7)	6.9 (5.8–8.2)	8.4 (7.8–9.2)	0.043
Widowed	2.4 (2.1–2.7)	2.8 (2.1–3.6)	2.3 (1.9–2.7)	0.269
Co‐habiting	17.9 (17.1–18.8)	14.2 (12.6–15.9)	19 (18.0–20.0)	< 0.001
Urban living area, % (95% CI)	84 (83.2–84.8)	93.8 (92.5–94.8)	81.2 (80.2–82.2)	< 0.001
Type of place of residence (by population size)^d^, % (95% CI)				
City	49.2 (48.2–50.3)	59.2 (56.9–61.5)	46.4 (45.1–47.6)	< 0.001
Town	34.8 (33.7–35.8)	34.5 (32.4–36.8)	34.8 (33.7–36.0)	0.837
Village	13.1 (12.4–13.9)	5.7 (4.7–6.9)	15.2 (14.4–16.1)	< 0.001
Place in the countryside that is not part of any city, town or village	2.9 (2.5–3.3)	0.5 (0.2–1.0)	3.6 (3.1–4.1)	< 0.001

*Note:* All reported findings are based on univariate analyses.

Abbreviation: CI, Confidence interval.

^a^ Southern European countries include Italy and Spain.

^b^ Northern, Western and Eastern European countries include Sweden, Romania, Belgium, France, Germany, the Netherlands, Poland and the United Kingdom.

^c^ BMI levels: Underweight: < 18.5 kg/m^2^; Normal weight: 18.5–24.9 kg/m^2^; Overweight: 25–29.9 kg/m^2^; Obesity: ≥ 30 kg/m^2^.

^d^ City ‐ more than 50,000 inhabitants; Town ‐ 2500 to 50,000 inhabitants; Village or small town ‐ less than 2500 inhabitants.

### 4.2 Prevalence of DGBI in Southern Europe

Of the total 54,127 participants included in the RFGES, 20,420 participants came from European countries and were subdivided into two main areas: 16,285 participants from Northern (*n* = 4111), Western (*n* = 8068), and Eastern (*n* = 4106) European countries and 4135 from Southern European countries (Table 2). In total, 44.0% (95% CI 42.4–45.5) of adults in Southern Europe met the criteria for at least one DGBI, significantly higher than the 39.0% (38.3–39.8) prevalence in Northern, Western, and Eastern European regions (*p* < 0.001). Southern Europe showed a greater prevalence across most DGBI categories (Table 1). Esophageal DGBI was reported by 8.3% of responders (95% CI 7.4–9.2) in Southern Europe compared to 5.7% (95% CI 5.3–6.1) in other European regions (*p* < 0.001). Gastroduodenal DGBI affected 12.5% (95% CI 11.5–13.5) in Southern Europe versus 9.6% (95% CI 9.2–10.1) elsewhere in Europe (*p* < 0.001). Specifically, the prevalence of FD was significantly higher in Southern Europe (8.2%, 95% CI 7.4–9.1) compared to other regions (6.9%, 95% CI 6.5–7.3) (*p* < 0.01). Similarly, both FD subtypes, postprandial distress syndrome and epigastric pain syndrome, were more frequent in Southern Europe. Bowel DGBI were the most prevalent, reported by 36.7% (95% CI 35.2–38.2) in Southern Europe versus 32.5% (95% CI 31.8–33.2) in other regions (*p* < 0.001). The prevalence of IBS was 4.6% (95% CI 4.0–5.3) in Southern Europe and 3.8% (95% CI 3.6–4.2) in other European regions (*p* < 0.05). Among IBS subtypes, IBS‐D (1.9% vs. 1.1%) and IBS‐C (1.5% vs. 1.2%) were modestly but significantly more common in Southern Europe (*p* < 0.001), while IBS‐M was substantially less common (1.1% vs. 1.3%; *p* < 0.001), and IBS‐U had similar frequencies (0.2% in both). FC was one of the most frequently reported DGBIs, affecting 13.2% (95% CI 12.1–14.2) in Southern Europe versus 11.0% (95% CI 10.5–11.5) in other European regions (*p* < 0.001). FDr, in contrast, was slightly less prevalent in Southern Europe (3.6% vs. 4.4%; *p* < 0.05). Southern Europe also had higher rates of anorectal DGBI overall (11.0% vs. 6.7%; *p* < 0.001), driven largely by a nearly double prevalence of proctalgia fugax (8.7% vs. 4.6%; *p* < 0.001). On average, individuals in Southern Europe tended to fulfill the criteria for a greater number of DGBI per person (∼0.8 vs. 0.6; *p* < 0.001) and more often had DGBI spanning multiple gastrointestinal regions, underscoring a higher rate of DGBI overlap. Analysis of all 22 DGBI across European regions showed consistently higher prevalence in Southern Europe, with broadly similar patterns in Northern and Western regions, supporting their aggregation into a single comparator group (Supporting Information S1).

**TABLE 2:** Prevalence rate (95% CI) for 22 DGBI and their overlaps among the Southern European countries, and Northern, Western, and Eastern European countries.

	Southern European countries^a^ (*n* = 4135)	Northern, Western and Eastern European countries^b^ (*n* = 16,285)	*p*‐value
Any DGBI	44.0 (42.4–45.5)	39.0 (38.3–39.8)	< 0.001
Any esophageal DGBI	8.3 (7.4–9.2)	5.7 (5.3–6.1)	< 0.001
Functional chest pain	1.5 (1.2–1.9)	1.5 (1.3–1.7)	> 0.999
Functional heartburn	1.5 (1.2–1.9)	0.9 (0.8–1.1)	< 0.001
Reflux hypersensitivity	1.5 (1.1–1.9)	0.7 (0.6–0.8)	< 0.001
Globus	1.1 (0.8–1.5)	0.8 (0.7–1.0)	0.046
Functional dysphagia	4.9 (4.2–5.6)	2.7 (2.5–3.0)	< 0.001
Any gastroduodenal DGBI	12.5 (11.5–13.5)	9.6 (9.2–10.1)	< 0.001
Functional dyspepsia	8.2 (7.4–9.1)	6.9 (6.5–7.3)	0.003
PDS	7.0 (6.2–7.8)	5.7 (5.3–6.1)	0.002
EPS	3.7 (3.1–4.3)	2.2 (2.0–2.5)	< 0.001
Belching disorder	1.3 (1.0–1.7)	0.7 (0.5–0.8)	< 0.001
Rumination syndrome	3.8 (3.3–4.5)	2.2 (2.0–2.5)	< 0.001
Chronic nausea/vomiting	1.1 (0.8–1.5)	0.9 (0.8–1.1)	0.329
Cyclic vomiting syndrome	2.0 (1.6–2.4)	0.9 (0.7–1.0)	< 0.001
Cannabinoid hyperemesis	0.0 (0.0–0.1)	0.0 (0.0–0.1)	> 0.999
Any bowel DGBI	36.7 (35.2–38.2)	32.5 (31.8–33.2)	< 0.001
Rome IV IBS	4.6 (4.0–5.3)	3.8 (3.6–4.2)	0.025
IBS‐C	1.5 (1.1–1.9)	1.2 (1.0–1.3)	< 0.001
IBS‐D	1.9 (1.5–2.3)	1.1 (1.0–1.3)	< 0.001
IBS‐M	1.1 (0.8–1.5)	1.3 (1.2–1.5)	< 0.001
IBS‐U	0.2 (0.1–0.3)	0.2 (0.2–0.3)	< 0.001
Functional constipation	13.2 (12.1–14.2)	11.0 (10.5–11.5)	< 0.001
Opioid‐induced constipation	1.2 (0.9–1.6)	1.3 (1.2–1.5)	0.535
Functional diarrhea	3.6 (3.1–4.2)	4.4 (4.‐4.7)	0.039
Functional abdominal bloating/distention	3.8 (3.3–4.5)	4.0 (3.7–4.3)	0.717
Unspecified functional bowel disorder	10.4 (9.5–11.4)	8.4 (8.0–8.9)	< 0.001
Centrally mediated abdominal pain syndrome	0.0 (0.0–0.1)	0.0 (0.0–0.1)	> 0.999
Functional biliary pain	0.1 (0.0–0.2)	0.1 (0.1–0.2)	> 0.999
Any anorectal DGBI	11.0 (10.0–12.0)	6.7 (6.4–7.1)	< 0.001
Fecal incontinence	1.3 (1.0–1.7)	1.5 (1.3–1.7)	0.379
Levator ani syndrome	1.4 (1.1–1.8)	1.1 (0.9–1.3)	0.101
Proctalgia fugax	8.7 (7.9–9.6)	4.6 (4.3–5.0)	< 0.001
Number of DGBIs, mean (95% CI)	0.8 (0.7–0.8)	0.6 (0.5–0.6)	< 0.001
Number of GI anatomic regions with DGBI^c^, mean (95% CI)	0.7 (0.6–0.7)	0.5 (0.5–0.6)	< 0.001
Number of GI anatomic regions with DGBI^c^			
One GI anatomic regions with DGBI	27.0 (25.7–28.4)	27.3 (26.6–28.0)	0.784
Two GI anatomic regions with DGBI	11.1 (10.2–12.1)	8.6 (8.2–9.0)	< 0.001
Three GI anatomic regions with DGBI	4.1 (3.5–4.7)	2.4 (2.1–2.6)	< 0.001
Four GI anatomic regions with DGBI	1.7 (1.3–2.1)	0.7 (0.6–0.9)	< 0.001

Abbreviations: CI, Confidence interval; DGBI, Disorder of the brain‐gut interaction; EPS, Epigastric pain syndrome; IBS‐C, Constipation predominant irritable bowel syndrome; IBS‐D, Diarrhea predominant irritable bowel syndrome; IBS‐M, Mixed irritable bowel syndrome; IBS‐U, Unspecified irritable bowel syndrome; PDS, Postprandial distress syndrome.

^a^ Southern European countries include Italy and Spain.

^b^ Northern, Western and Eastern European countries include Sweden, Romania, Belgium, France, Germany, the Netherlands, Poland and the United Kingdom.

^c^ One patient can have multiple DGBIs in the same region; GI anatomic regions are: esophageal, gastroduodenal, bowel, and anorectal.

### 4.3 Psychological Distress, Somatic Symptom Burden and Quality of Life

Psychological burden and somatic symptomatology were highly prevalent among individuals with at least one DGBI and showed significant regional variation across Europe (Supporting Information S1). Overall, more than half of the population reported the presence of anxiety and/or depressive symptoms (58.8%), with a mean anxiety and depression score of 3.8. Participants with at least one DGBI from Southern European countries exhibited a significantly higher prevalence of psychological distress compared with those from Northern, Western, and Eastern Europe (68.9% vs. 55.8%, *p* < 0.001), as well as higher mean psychological distress scores (4.2 vs. 3.6, *p* < 0.001).

Somatic symptom burden, assessed via the PHQ‐12 score, was considerable in the overall cohort, with a mean somatic symptom scale score of 6.6 (Supporting Information S1). Notably, individuals from Southern Europe reported a significantly lower somatic symptom score than those from other European regions (6.3 vs. 6.7, *p* < 0.001). Several extra‐intestinal symptoms demonstrated marked geographic variability. Participants from Southern Europe more frequently reported pain or problems during sexual intercourse, headaches, and menstrual‐related symptoms, while fatigue and low energy, as well as sleep disturbances, were more commonly reported in Northern, Western, and Eastern European countries.

In terms of general health‐related quality of life, scores by the PROMIS‐10 questionnaire, showed comparable results between Southern European participants and the rest of Europe, with slight differences in the physical health scores, which higher in the former group (mean 13.8 vs. 13.3, *p* < 0.001). (Supporting Information S1) Furthermore, daily functioning and productivity were considerably affected across the entire cohort, even though individuals from Southern Europe reported significantly lower work productivity loss and activity impairment (*p* = 0.003).

### 4.4 Healthcare Utilization Patterns

Healthcare‐seeking behavior differed substantially by region (Supporting Information S1). In fact, 56.7% of participants from Southern Europe had consulted a physician for bowel‐related problems, compared with 41.7% in other European countries (*p* < 0.001). Southern European participants reported more frequent medical consultations and a higher use of medical care and medications for constipation, nausea, heartburn, and bloating. In contrast, antidepressant use was more common in the other European countries.

### 4.5 Dietary Habits and Mediterranean Diet Adherence

Dietary patterns varied significantly across European regions (Supporting Information S1). In fact, Southern European participants reported lower intake of meat and higher consumption of fish, fruits, pasta, bread, and rice. In contrast, participants from other European regions consumed more vegetables and legumes. Food avoidance was reported exclusively by Italian participants and affected nearly one‐third of respondents within that subgroup.

### 4.6 Factors Associated With DGBI

The multivariate logistic regression analyses identified independent sociodemographic, clinical, psychosocial, and healthcare access factors associated with the presence of any DGBI, as well as with specific diagnoses of IBS and FD (Table 3). Living in Southern European countries was associated with a higher likelihood of reporting any DGBI compared with residence in Northern, Western, and Eastern Europe (OR 1.13, 95% CI 1.04–1.22). Younger age (18–39 years) was also an independent predictor (OR 1.11, 95% CI 1.04–1.18), as was female sex, which conferred a 36% increased odds of having any DGBI (OR 1.36, 95% CI 1.28–1.45). Years of education showed a modest but statistically significant positive association with DGBI (OR 1.01, 95% CI 1.00–1.01). Psychosocial factors demonstrated the strongest associations. Both higher somatic symptom burden assessed via PHQ‐12 scores and the presence of anxiety and/or depressive symptoms were consistently associated with higher odds of any DGBI (somatic symptom burden: OR 1.18 per PHQ‐12 score unit increase, 95% CI 1.17–1.20; anxiety and/or depression: OR 1.85, 95% CI 1.73–1.98). In addition, living in an urban area was modestly associated with the presence of any DGBI (OR 1.10, 95% CI 1.01–1.19). Healthcare access, defined as physician visits occurring once per month or more, was also independently associated with higher odds of DGBI (OR 1.16, 95% CI 1.05–1.28).

**TABLE 3:** Multivariate analysis assessing factors associated with any DGBIs, IBS and functional dyspepsia occurrence in Europe.

	Any DGBI		IBS		Functional dyspepsia	
	OR (95% CI)	*p*‐value	OR (95% CI)	*p*‐value	OR (95% CI)	*p*‐value
Living in Southern European countries^a^	1.13 (1.04–1.22)	0.003	1.19 (1.00–1.43)	0.048	1.19 (1.03–1.36)	0.015
Age (18–39 years)	1.11 (1.04–1.18)	0.001	1.41 (1.21–1.64)	< 0.001	1.29 (1.16–1.46)	< 0.001
Gender (female)	1.36 (1.28–1.45)	< 0.001	1.30 (1.11–1.52)	0.001	NS	NS
Somatic symptoms scale^b^	1.18 (1.17–1.20)	< 0.001	1.23 (1.21–1.26)	< 0.001	1.21 (1.19–1.22)	< 0.001
Living in urban area	1.10 (1.01–1.19)	0.028	NS	NS	NS	NS
Presence of anxiety and depression (mild, moderate, severe)^c^	1.85 (1.73–1.98)	< 0.001	2.58 (2.14–3.11)	< 0.001	2.07 (1.82–2.36)	< 0.001
Healthcare access^d^	1.16 (1.05–1.28)	0.003	1.42 (1.18–1.71)	< 0.001	1.56 (1.35–1.80)	< 0.001
Education (years)	1.01 (1.00–1.01)	0.044	1.02 (1.00–1.03)	0.009	NS	NS

Abbreviations: CI, Confidence interval; IBS, Irritable bowel syndrome; NS, Not significant; OR, Odd‐ratio.

^a^ Southern European countries include Italy and Spain.

^b^ Somatic symptoms were evaluated by PHQ12 without menstrual item.

^c^ Anxiety and depression were evaluated by PHQ‐4 questionnaire, while 0‐2 score relates to non‐psychological distress, 3‐5 score = mild psychological distress, 6‐8 score = moderate psychological distress, and 9‐12 score = severe psychological distress.

^2^Somatic symptom scale score was evaluated by the PHQ‐12 questionnaire, without 3 gastrointestinal items and a menstrual symptom item.

^d^ Defined as physician visits occurring at a frequency of once per month or more.

As for IBS risk, living in Southern Europe was also an independent predictor of IBS (OR 1.19, 95% CI 1.00–1.43), as well as younger age (OR 1.41, 95% CI 1.21–1.64), female sex (OR 1.30, 95% CI 1.11–1.52), somatic symptom burden (OR 1.23 per PHQ‐12 score unit increase, 95% CI 1.21–1.26), presence of anxiety and/or depression (OR 2.58, 95% CI 2.14–3.11), greater healthcare access (or 1.42, 95% CI 1.18–1.71), and higher educational attainment (OR 1.02, 95% CI 1.00–1.03).

In the multivariate analysis assessing factors associated with FD, a similar pattern was observed. Residence in Southern European countries was again independently associated with increased odds of having FD (OR 1.19, 95% CI 1.03–1.36). Younger age remained a significant predictor (OR 1.29, 95% CI 1.16–1.46), as well as higher somatic symptom scores (OR 1.21, 95% CI 1.19–1.22), presence of anxiety and/or depression (OR 2.07, 95% CI 1.82–2.36), and greater healthcare access (OR 1.56, 95% CI 1.35–1.80). Female sex, education and urban residence were not significantly associated with functional dyspepsia.

## 5 Discussion

The internet survey of the RFGES provides a uniquely large and multi‐national perspective on DGBI, encompassing over 54,000 adults across 26 countries around the world [9]. This study reveals a notably higher prevalence of DGBI in Southern Europe compared to other European regions. Approximately 44% of adults in Southern Europe (Italy and Spain) met the criteria for at least one DGBI, versus ∼39% in Northern, Western, and Eastern Europe. These figures are in line with, or slightly above, the prevalence reported in the RFGES, which found that about 40% of respondents worldwide have a DGBI [9]. The comparison between Southern Europe and the rest of Europe was designed to provide a macro‐level epidemiological contrast across the continent. To achieve this, Northern, Western, and Eastern European countries were grouped into a single comparator, in line with established geopolitical classifications. To address potential heterogeneity, additional analyses comparing Northern, Western, Eastern, and Southern Europe were performed. These analyses demonstrated a consistent gradient, with Southern Europe exhibiting the highest prevalence rates across most DGBI categories, Northern and Western Europe the lowest, and Eastern Europe generally showing intermediate values that remained lower than those observed in Southern Europe for nearly all DGBI categories, supporting the use of a combined comparator group in the primary analyses. Several lifestyle and clinical factors were evaluated in relation to DGBI. Notably, although BMI was not independently associated with DGBI in multivariable analyses, regional differences were observed within the DGBI population. Specifically, individuals with DGBI in Northern, Western, and Eastern Europe had higher BMI and a greater prevalence of obesity compared with those in Southern Europe. A recently published report from the RFGES thoroughly described the association between overweight and obesity and DGBI in Europe, finding that these conditions are often linked, especially in females, patients with higher level of psychological distress and more with severe non‐gastrointestinal somatic symptoms [24]. However, our results show that obesity rates in patients with DGBI were significantly higher in Northern, Western and Eastern Europe compared with Southern Europe. Therefore, the directionality of the association between obesity and DGBI is still unclear. It is possible that the higher somatic symptom burden of DGBI found in Northern, Western and Eastern Europe may lead to reduced levels of physical activity and a less regulated dietary pattern, which could increase the risk of obesity. In fact, diets high in fat, processed food, and refined carbohydrates are often consumed in the context of psychological and physical distress. In turn, a Western diet can promote gut microbiome alterations and low‐grade inflammation, thereby contributing to DGBI pathogenesis. [6, 25, 26, 27]. Nonetheless, our results show higher rates of DGBI in countries where traditionally the Mediterranean diet is more common. The Mediterranean diet, rich in fiber, fruits, vegetables, fish, and olive oil, has been consistently associated with numerous health benefits, including anti‐inflammatory effects and favorable gut microbiota modulation. Recent cross‐sectional studies have shown that high adherence to the Mediterranean diet is linked to lower psychological distress and better sleep, which may indirectly protect against DGBI [27, 28, 29]. Furthermore, a systematic review and network metanalysis on the efficacy of different diets in IBS found that adherence to the Mediterranean diet was associated with lower rates of overall IBS symptoms [27]. Evidence from two randomized control studies [13, 17] in 85 patients showed an odds ratio of 0.27 (95% CI 0.11–0.67) for IBS symptom reduction. Nonetheless, it should be considered that adherence to this dietary pattern may vary substantially within regions. In our cohort, urban residence was markedly higher in Southern Europe, and it is plausible that the protective effects of a traditional Mediterranean lifestyle may be attenuated in urban settings, where dietary habits are often more Westernized and lifestyle factors such as stress and reduced physical activity are more prevalent In this context, the expected benefits of the Mediterranean diet may be less evident, particularly compared with more rural environments where traditional dietary patterns are better preserved. Moreover, it should be acknowledged that certain components of the Mediterranean diet, such as fruits, legumes, and wheat are high in fermentable carbohydrates (FODMAPs), which can provoke symptoms in sensitive individuals with IBS [30]. Consistently, nearly one‐third of individuals in Italy reported food avoidance, suggesting that symptom‐driven dietary modification is common in this population and may reflect increased awareness of food‐related triggers, likely explaining why in the overall cohort of patients with DGBI a higher consumption of legumes and vegetables was found in non‐Southern European countries. Indeed, a randomized controlled trial of 106 IBS patients demonstrated that while adherence to a standard Mediterranean diet was not associated with overall IBS symptom severity compared with a healthy population, certain foods within the diet were linked to increased symptoms [31]. These findings suggest that the standard Mediterranean diet may not be suitable for all patients with IBS and likely requires personalization in those with heightened sensitivity. Future work should clarify how regional culture, diet, and environment interact to shape the observed gradient in DGBI prevalence.

Our study confirms a strong association between psychological distress and DGBI, reinforcing the biopsychosocial model of these disorders. In fact, both the presence of anxiety and depression and higher somatic symptom score were significantly associated with higher rates of DGBI, such as IBS and FD, in multivariate analysis. Moreover, our results show that Southern European individuals with DGBI more commonly had comorbid depression and anxiety with respect to Northern, Western and Eastern Europeans, associated with the higher rates of DGBI found in this population. These results mirror the evidence linking IBS and other DGBI with anxiety, depression, and other psychosocial factors [12, 32]. Not only can anxiety and depression exacerbate gastrointestinal symptoms via stress‐related mechanisms (e.g., hypothalamic‐pituitary axis activation, autonomic dysfunction), but chronic gastrointestinal symptoms can reciprocally worsen mental health, creating a vicious cycle on the gut–brain axis. Interestingly, there is a discrepancy between prevalence and burden: although DGBI are more frequently reported in Southern Europe, their overall impact, measured by somatic symptom scores and work and activity impairment, appears greater in other European regions. This pattern may suggest differences in symptom perception and reporting, with individuals in Southern Europe potentially more likely to report milder symptoms that do not strongly affect daily functioning, even in the context of higher psychological distress. Conversely, in Northern, Western, and Eastern Europe, DGBI may be reported less frequently but, when present, may tend to have a greater impact on daily life and functioning.

We also found that somatic symptom burden beyond gastrointestinal symptoms was elevated in those with DGBI both in Southern Europe and the rest of the continent [8]. This overlap has been documented in previous studies; for example, fibromyalgia [33, 34], chronic fatigue syndrome [35], and functional gastrointestinal disorders [8] frequently co‐occur, suggesting a shared pathophysiological mechanism such as central nervous system hypervigilance or dysregulation of pain modulation [36, 37]. Our data reinforce the theory that DGBI rarely exists in isolation and that patients often present with a mosaic of symptoms beyond the gastrointestinal tract. This has practical implications: clinicians should screen DGBI patients for extra‐intestinal symptoms and conditions and, conversely, patients presenting with multisystem somatic complaints should be screened for underlying DGBI. Notably, the association with somatic symptoms remained significant in multivariate analysis, suggesting that this relationship is not fully accounted for by co‐occurring psychological comorbidity.

Our study has several limitations. The RFGES used internet‐based questionnaires, which might under‐represent the elderly population or those without internet access. Self‐reporting of diet and symptoms can introduce recall bias. Moreover, it cannot be excluded that differences in healthcare systems and cultural differences in symptom reporting and disease awareness across regions may have contributed to higher reporting of DGBIs, as suggested by the greater health‐seeking behavior observed in Southern European countries. Nevertheless, the use of validated Rome IV modules, the large and representative sample, and the consistency with established epidemiology reinforce the findings and are important strengths. Furthermore, it should be acknowledged that this manuscript relies on regional data, which prevents drawing conclusions about individual risk factors. A further limitation concerns the aggregation of data from Northern, Western, and Eastern Europe into a single category, which may mask significant relevant regional heterogeneity, even though additional analyses were performed to reduce this limitation. Moreover, Southern Europe was represented by only two countries (Italy and Spain), potentially limiting the external validity and generalizability of the results. An additional limitation is the lack of procedures such as endoscopy or manometry; therefore, a small proportion of patients classified as DGBI may have underlying “organic” diseases. However, since the methods of data acquisition were the same both within Italy and within Europe, this does not likely modify the differences observed between populations. Lastly, data on pathophysiological mechanisms, such as genetics, permeability evaluations, microbiota analysis and neuro‐immune interactions, would have been useful to provide pathophysiological backgrounds to the results that were obtained.

In conclusion, Southern Europe has a higher prevalence of DGBI, driven by a complex nexus of gender, age, psychological, somatization, and possibly dietary factors. These results challenge the notion that a Mediterranean population may be less affected by DGBI. It is possible that economic or societal stressors in Southern Europe have increased psychological distress in the population, which in turn fuels DGBI. Nonetheless, individuals with DGBI in Southern Europe display lower somatic symptom burden and functional impairment, indicating a dissociation between prevalence and overall disease burden. Future research should build on this by investigating causal pathways via longitudinal studies on genetic polymorphisms, diet quality, other pathophysiological evaluations and socioeconomical analysis in relation to DGBI onset.

## Author Contributions

Marasco G, Hod K and Barbara G designed the study. Hod K performed statistical analysis. All authors critically discussed the study results. Hod K, Marasco G, Colecchia L and Barbara G drafted the manuscript. All authors critically revised and approved the final version of the manuscript, including the authorship list.

## Funding

The authors have nothing to report.

## Ethics Statement

The Rome Foundation Global Epidemiology Study received ethics approval or was deemed exempt by the appropriate institutional review boards or ethics committees in each participating country. The study was conducted in accordance with the Declaration of Helsinki and applicable regulatory requirements.

## Consent

All participants provided electronic informed consent prior to participation in the survey, and all data were collected anonymously.

## Conflicts of Interest

The authors declare no conflicts of interest.

## Permission to Reproduce Material From Other Sources

The authors have nothing to report.

## Supporting information


Supporting Information S1

## Data Availability

The data that support the findings of this study are available from the corresponding author upon reasonable request.
