# Correction to “Comparative Study on Burden, Features and Determinants of Disorders of Gut‐Brain Interaction Between Southern Europe and the Rest of Continent: Results From the Rome Foundation Global Epidemiology Study”

**DOI:** 10.1002/ueg2.70252

**Published:** 2026-06-29

G. Marasco, K. Hod, L. Colecchia, et al., “Comparative Study on Burden, Features and Determinants of Disorders of Gut‐Brain Interaction Between Southern Europe and the Rest of Continent: Results From the Rome Foundation Global Epidemiology Study,” *United European Gastroenterology Journal* 14, no. 4 (2026): e70226, https://doi.org/10.1002/ueg2.70226.

On page 1 in the Abstract, the sentence “Irritable bowel syndrome and functional dyspepsia were more prevalent in Southern Europe than in the rest of Europe. Similar trends were found for functional constipation and functional diarrhea.” has been modified in “Irritable bowel syndrome, functional dyspepsia, and functional constipation were more prevalent in Southern Europe than in the rest of Europe.” On page 4, in the Results section, the citation to Table 1 for prevalence estimates has been corrected to Table 2. In Table 2, the confidence interval for functional diarrhea in Northern, Western, and Eastern European countries was incorrectly reported as “4.‐4.7”, and has been corrected to: “4.1–4.7”. On page 9, in the Results section, the sentence: “Analysis of all 22 DGBI across European regions showed consistently higher prevalence in Southern Europe” has been revised to: “Analysis of all 22 DGBI across European regions showed that most DGBI tended to have higher prevalence estimates in Southern Europe, while Northern and Western regions demonstrated broadly similar patterns, supporting their aggregation into a single comparator group.”

In Table 2, *p*‐values relating to IBS subtypes were incorrect and have now been corrected as follows: IBS‐C: *p* = 0.093, IBS‐M: *p* = 0.247, IBS‐U: *p* = 0.577. Accordingly, the corresponding Results text was revised to (page 4): “Among IBS subtypes, IBS‐D was modestly but significantly more common in Southern Europe (1.9% vs. 1.1%, *p* < 0.001), whereas IBS‐C tended to be more prevalent in Southern Europe without reaching statistical significance (1.5% vs. 1.2%; *p* = 0.093). IBS‐M and IBS‐U had similar frequencies across regions (1.1% vs. 1.3%; *p* = 0.247, and 0.2% in both groups, respectively).”

In Table 3 and throughout the manuscript, the somatic symptom burden scale was inconsistently described. The questionnaire was originally referred to as “PHQ‐12,” whereas the actual scale used was a modified version of PHQ‐15 without three gastrointestinal symptom items and one menstrual symptom item. Accordingly, all relevant sections were corrected.

Reference 10 contained inaccuracies in the author list and DOI and has been corrected as follows: “Arias‐de la Torre J, Vilagut G, Ronaldson A, Bakolis I, et al. Prevalence and variability of depressive symptoms in Europe: update using representative data from the second and third waves of the European Health Interview Survey (EHIS‐2 and EHIS‐3). Lancet Public Health. 2023; 8–98. doi:10.1016/S2468‐2667(2300220‐7.”) The citation for IBM SPSS Statistics incorrectly stated (page 3): “IBM Corp. Released 2019” and has been corrected to: “IBM Corp. Released 2024.”

The authors sincerely apologize for these errors and appreciate the opportunity to correct the scientific record.

