# Supplementary material for: Comparative Study on Burden, Features and Determinants of Disorders of Gut‐Brain Interaction Between Southern Europe and the Rest of Continent: Results From the Rome Foundation Global Epidemiology Study
Source: United European Gastroenterol J. 2026 May 5;14(4):e70226. doi: 10.1002/ueg2.70226 (PMC13139900; doi:10.1002/ueg2.70226)
Supplement: Supplementary file 1 — Supporting Information S1 [file UEG2-14-e70226-s001.docx]

**Supplementary Table 1. Prevalence rate (95% CI) for 22 DGBI among the Southern, Northern, Western, and Eastern European countries**

| **Outcome** | **Southern Europe** | **Northern Europe** | **Western Europe** | **Eastern Europe** | **Overall p-value** |
| --- | --- | --- | --- | --- | --- |
| Any DGBI | 44.0% (42.5-45.5)^a^ | 37.8% (36.4-39.3)^b^ | 37.5% (36.5-38.6)^b^ | 43.1% (41.6-44.6)^a^ | <0.001 |
| Any esophageal DGBI | 8.3% (7.5-9.1)^a^ | 6.8% (6.1-7.6)^b^ | 5.3% (4.8-5.8)^c^ | 5.4% (4.8-6.1)^c^ | <0.001 |
| Functional chest pain | 1.5% (1.2-1.9)^a^ | 1.7% (1.3-2.1)^a^ | 1.5% (1.2-1.8)^a^ | 1.5% (1.1-1.9)^a^ | 0.886 |
| Functional heartburn | 1.5% (1.2-1.9)^a^ | 0.9% (0.7-1.3)^ab^ | 0.9% (0.7-1.1)^b^ | 0.9% (0.6-1.2)^ab^ | 0.008 |
| Reflux hypersensitivity | 1.5% (1.1-1.9)^a^ | 0.8% (0.5-1.1)^b^ | 0.6% (0.5-0.8)^b^ | 0.7% (0.5-1.0)^b^ | <0.001 |
| Globus | 1.1% (0.9-1.5)^a^ | 1.1% (0.8-1.5)^a^ | 0.7% (0.6-1.0)^a^ | 0.6% (0.4-0.9)^a^ | 0.011 |
| Functional dysphagia | 4.9% (4.2-5.6)^a^ | 3.3% (2.8-3.9)^b^ | 2.3% (2.0-2.7)^c^ | 2.8% (2.4-3.4)^bc^ | <0.001 |
| Any gastroduodenal DGBI | 12.5% (11.5-13.5)^a^ | 10.4% (9.5-11.3)^b^ | 8.7% (8.1-9.3)^c^ | 10.7% (9.8-11.7)^b^ | <0.001 |
| Functional dyspepsia | 8.2% (7.4-9.1)^a^ | 7.4% (6.6-8.2)^a^ | 6.1% (5.6-6.7)^b^ | 7.8% (7.1-8.7)^a^ | <0.001 |
| PDS | 7.0% (6.3-7.8)^a^ | 6.0% (5.3-6.8)^ab^ | 5.0% (4.5-5.5)^b^ | 6.8% (6.1-7.6)^a^ | <0.001 |
| EPS | 3.7% (3.1-4.3)^a^ | 2.3% (1.9-2.8)^b^ | 2.0% (1.8-2.4)^b^ | 2.5% (2.1-3.0)^b^ | <0.001 |
| Belching disorder | 1.3% (1.0-1.7)^a^ | 0.6% (0.4-0.9)^b^ | 0.4% (0.3-0.6)^b^ | 1.2% (0.9-1.6)^a^ | <0.001 |
| Rumination syndrome | 3.8% (3.3-4.4)^a^ | 2.7% (2.2-3.2)^b^ | 2.1% (1.8-2.4)^b^ | 2.0% (1.6-2.5)^b^ | <0.001 |
| Chronic nausea/vomiting | 1.1% (0.8-1.5)^a^ | 1.1% (0.8-1.5)^a^ | 0.8% (0.7-1.1)^a^ | 0.9% (0.7-1.3)^a^ | 0.438 |
| Cyclic vomiting syndrome | 2.0% (1.6-2.4)^a^ | 0.5% (0.3-0.8)^b^ | 0.8% (0.6-1.0)^c^ | 1.3% (1.0-1.7)^d^ | <0.001 |
| Cannabinoid hyperemesis | 0.0% (0.0-0.1)^a^ | 0.1% (0.0-0.2)^a^ | 0.0% (0.0-0.1)^a^ | 0.0% (0.0-0.2)^a^ | 0.606 |
| Any bowel DGBI | 36.7% (35.2-38.1)^a^ | 30.5% (29.1-31.9)^b^ | 31.7% (30.7-32.7)^b^ | 36.0% (34.5-37.5)^a^ | <0.001 |
| Rome IV IBS | 4.6% (4.0-5.3)^a^ | 4.0% (3.4-4.6)^a^ | 3.7% (3.3-4.2)^a^ | 3.9% (3.4-4.6)^a^ | 0.120 |
| Functional constipation | 13.2% (12.2-14.2)^a^ | 9.3% (8.4-10.2)^b^ | 10.9% (10.3-11.6)^c^ | 12.8% (11.8-13.9)^a^ | <0.001 |
| Opioid-induced constipation | 1.2% (0.9-1.6)^a^ | 1.7% (1.3-2.1)^a^ | 1.2% (1.0-1.5)^a^ | 1.2% (0.9-1.5)^a^ | 0.156 |
| Functional diarrhea | 3.6% (3.1-4.2)^ab^ | 5.0% (4.4-5.7)^c^ | 4.5% (4.1-5.0)^ac^ | 3.4% (2.9-4.0)^b^ | <0.001 |
| Functional abdominal bloating/distention | 3.8% (3.3-4.4)^a^ | 3.5% (3.0-4.1)^a^ | 3.2% (2.8-3.6)^a^ | 6.0% (5.3-6.8)^b^ | <0.001 |
| Unspecified functional bowel disorder | 10.4% (9.6-11.4)^a^ | 7.4% (6.7-8.3)^b^ | 8.4% (7.8-9.0)^bc^ | 9.5% (8.6-10.4)^ac^ | <0.001 |
| Centrally mediated abdominal pain syndrome | 0.0% (0.0-0.1)^a^ | 0.0% (0.0-0.1)^a^ | 0.0% (0.0-0.1)^a^ | 0.0% (0.0-0.1)^a^ | 0.923 |
| Functional biliary pain | 0.1% (0.0-0.2)^a^ | 0.1% (0.1-0.3)^a^ | 0.1% (0.1-0.2)^a^ | 0.0% (0.0-0.2)^a^ | 0.687 |
| Any anorectal DGBI | 11.0% (10.1-12.0)^a^ | 6.9% (6.2-7.7)^b^ | 5.9% (5.4-6.4)^c^ | 8.3% (7.5-9.2)^d^ | <0.001 |
| Fecal incontinence | 1.3% (1.0-1.7)^a^ | 2.2% (1.8-2.7)^b^ | 1.3% (1.1-1.6)^a^ | 1.1% (0.8-1.5)^a^ | <0.001 |
| Levator ani syndrome | 1.4% (1.1-1.8)^a^ | 1.3% (1.0-1.7)^a^ | 1.1% (0.9-1.3)^a^ | 0.9% (0.7-1.3)^a^ | 0.109 |
| Proctalgia fugax | 8.7% (7.9-9.6)^a^ | 4.0% (3.5-4.7)^b^ | 3.9% (3.5-4.3)^b^ | 6.7% (6.0-7.5)^c^ | <0.001 |

Pairwise comparisons were adjusted for multiple testing using the Holm method.

Groups sharing the same superscript letter are not significantly different (p > 0.05).

Abbreviations: CI, Confidence interval; DGBI, Disorder of the brain-gut interaction; EPS, Epigastric pain syndrome; IBS-C, Constipation predominant irritable bowel syndrome; IBS-D, Diarrhea predominant irritable bowel syndrome; IBS-M, Mixed irritable bowel syndrome; IBS-U, Unspecified irritable bowel syndrome; PDS, Postprandial distress syndrome

Southern European countries include Italy and Spain

Northern European countries include Sweden, and United Kingdom

Western European countries include Belgium, France, Germany, and Netherlands

Eastern European countries include Romania, and Poland

**Supplementary table 2. Psychological distress, extra-intestinal comorbidity and general assessments of the European population with at least one DGBI**

|  | Overall European population with at least one DGBI (n=8,170) | Southern European countries^1^ with at least one DGBI (n=1,818) | Northern, Western and Eastern European countries^2^ with at least one DGBI (n=6,352) | P-value |
| --- | --- | --- | --- | --- |
| **General assessments** |  |  |  |  |
| - - Global physical health (PROMIS Global-10), mean (95% CI) | 13.4 (13.4–13.5) | 13.8 (13.6–13.9) | 13.3 (13.3–13.4) | <0.001 |
| - - Global mental health (PROMIS Global-10), mean (95% CI) | 12.7 (12.6–12.8) | 12.8 (12.6–12.9) | 12.7 (12.6–12.8) | 0.304 |
| **Somatic symptoms** |  |  |  |  |
| - - Somatic symptoms scale score^3^, mean (95% CI) | 6.6 (6.6–6.7) | 6.3 (6.1–6.5) | 6.7 (6.6–6.8) | <0.001 |
| - - Fibromyalgia syndrome^4^, % (95% CI) | 3.1 (2.7–3.5) | 3.2 (2.4–4.1) | 3.1 (2.6–3.5) | 0.762 |
| - - Pain or problems during sexual intercourse^5^, % (95% CI) | 16.4 (15.6–17.2) | 18.3 (16.5–20.0) | 15.9 (15.0–16.8) | 0.016 |
| - - Feeling tired or having low energy^5^, % (95% CI) | 78.7 (77.9–79.6) | 72.1 (70.0–74.1) | 80.7 (79.7–81.6) | <0.001 |
| - - Trouble sleeping^5^, % (95% CI) | 65.3 (64.3–66.4) | 62.9 (60.7–65.1) | 66.0 (64.8–67.2) | 0.016 |
| - - Headaches^5^, % (95% CI) | 65.8 (64.8–66.8) | 68.1 (66.0–70.2) | 65.2 (64.0–66.3) | 0.020 |
| - - Arms, legs, or joints pains^5^, % (95% CI) | 67.9 (66.9–68.9) | 66.4 (64.2–68.6) | 68.3 (67.2–69.5) | 0.126 |
| - - Menstrual cramps or problems with period (women only)^5^, % (95% CI) | 70.9 (69.4–72.4) | 76.9 (74.0–79.7) | 69.1 (67.3–70.8) | <0.001 |
| **Psychological variables** |  |  |  |  |
| - - Presence of anxiety and depression^6^, % (95% CI) | 58.8 (57.7–59.8) | 68.9 (66.8–71.0) | 55.8 (54.6–57.1) | <0.001 |
| - - Anxiety and depression score^6^, mean (95% CI) | 3.8 (3.7–3.8) | 4.2 (4.1–4.4) | 3.6 (3.5–3.7) | <0.001 |
| **Concerns and embarrassment regarding bowel functioning** |  |  |  |  |
| - - Concern regarding bowel functioning (somewhat/very concerned), % (95% CI) | 62.0 (60.9–63.0) | 61.9 (59.7–64.2) | 62.0 (60.8–63.2) | 0.994 |
| - - Embarrassment discussing bowel function with family or friends, % (95% CI) | 47.1 (46.0–48.2) | 43.6 (41.3–45.8) | 48.1 (46.9–49.3) | 0.001 |
| - - Stress, pressure or tension affecting bowel functioning, % (95% CI) | 5.2 (74.2–76.1) | 77.3 (75.4–79.3) | 74.6 (73.5–75.6) | 0.017 |
| **Work productivity and activity impairment^7^, mean (95% CI)** |  |  |  |  |
| - - % Work time missed due to health | 7.3 (6.5–8.1) | 5.9 (4.8–7.0) | 8.2 (7.1–9.3) | 0.003 |
| - - % Impairment while working due to health | 15.3 (14.5–16.2) | 14.5 (13.2–15.8) | 15.9 (14.8–17.0) | 0.110 |
| - - % Overall work impairment due to health | 20.5 (19.5–21.6) | 18.5 (16.9–20.1) | 21.8 (20.4–23.2) | 0.003 |
| - - % Activity impairment due to health | 24.9 (24.1–25.7) | 21.1 (19.9–22.3) | 27.0 (26.0–28.0) | <0.001 |

CI, Confidence interval

^1^Southern European countries include Italy and Spain

^2^Northern, Western and Eastern European countries include Sweden, Romania, Belgium, France, Germany, Netherlands, Poland and United Kingdom

^3^Somatic symptoms were evaluated using a modified PHQ-15 without three gastrointestinal symptom items and one menstrual symptom item.

^4^Fibromyalgia syndrome was assessed by the question: "Have you ever been diagnosed by a doctor as having Fibromyalgia?"

^5^Pain or problems during sexual intercourse, feeling tired or having low energy, trouble sleeping, headaches, legs or joints pain, menstrual cramps or problems with period were evaluated by the following questions: In the past 4 weeks, how much have you been bothered by any of the following problems?" followed by the relevant symptoms, with menstrual cramps or problems with period being applied for women only.

^6^Anxiety and depression were evaluated by PHQ-4; presence of anxiety and depression was defined as any level of these disorders (i.e. mild, moderate and severe), as 0-2 score relates to none psychological distress, 3-5 score = mild psychological distress, 6-8 score = moderate psychological distress, and 9-12 score = severe psychological distress.

^7^Work productivity and activity impairment was assessed by WPAI Scoring:

- Percent work time missed due to health: Q2/(Q2+Q4)
- Percent impairment while working due to health: Q5/10
- Percent activity impairment due to health: Q6/10
- Percent overall work impairment due to health: Q2 / (Q2 + Q4) + [1 – Q2 / (Q2 + Q4)] × (Q5 / 10)

Q2: "In the last seven days, how many hours were you away from work because of your health problems?"

Q4: "In the past seven days, how many hours did you work?"

Q5: "During the past seven days, to what extent did your health problems affect your productivity while you were working?"

Q6: "During the past seven days, to what extent did your health problems affect your ability to carry out your everyday activities other than work?"

All reported findings are based on univariate analyses.

**Supplementary table 3. General and bowel related healthcare usage of the European population with at least one DGBI**

|  | Overall European population with at least one DGBI (n=8,170) | Southern European countries^1^ with at least one DGBI (n=1,818) | Northern, Western and Eastern European countries^2^ with at least one DGBI (n=6,352) | P-value |
| --- | --- | --- | --- | --- |
| Ever visited doctor for bowel problem, % (95% CI) | 45.0% (44.0–46.1) | 56.7% (54.4–58.9) | 41.7% (40.5–42.9) | <0.001 |
| Frequency of doctor visits, % (95% CI): | | | | |
| - - Never | 1.8% (1.5–2.1) | 1.3% (0.8–1.8) | 1.9% (1.6–2.3) | 0.071 |
| - - Less than once a year | 15.4% (14.7–16.2) | 10.6% (9.2–12.0) | 16.8% (15.9–17.7) | <0.001 |
| - - Once a year | 13.7% (13.0–14.5) | 9.5% (8.2–10.9) | 14.9% (14.1–15.8) | <0.001 |
| - - A few times a year | 53.7% (52.7–54.8) | 59.5% (57.3–61.8) | 52.1% (50.9–53.3) | <0.001 |
| - - Once a month or more | 15.3% (14.5–16.1) | 19.1% (17.3–20.9) | 14.2% (13.3–15.1) | <0.001 |
| Type of healthcare usages, % (95% CI): | | | | |
| - - Western style medicine^3^ | 44.8% (43.7–45.9) | 56.5% (54.3–58.8) | 41.5% (40.2–42.7) | <0.001 |
| - - Traditional / folk healer^4^ | 2.0% (1.7–2.4) | 1.7% (1.1–2.3) | 2.1% (1.8–2.5) | 0.287 |
| Medications taken regularly (at least once a week), % (95% CI) | | | | |
| - - Medications for constipation | 11.4% (10.7–12.1) | 15.2% (13.5–16.8) | 10.3% (9.5–11.0) | <0.001 |
| - - Medications for diarrhea | 7.0% (6.4–7.5) | 7.6% (6.4–8.9) | 6.8% (6.2–7.4) | 0.223 |
| - - Medications for nausea | 5.7% (5.2–6.2) | 7.1% (5.9–8.3) | 5.3% (4.7–5.8) | 0.003 |
| - - Medications for heartburn or to reduce stomach acid | 27.8% (26.8–28.7) | 35.3% (33.1–37.5) | 25.6% (24.5–26.7) | <0.001 |
| - - Medications for gas or bloating | 12.4% (11.7–13.1) | 16.9% (15.2–18.7) | 11.1% (10.4–11.9) | <0.001 |
| - - Medications for depression | 14.2% (13.5–15.0) | 12.0% (10.5–13.5) | 14.8% (14.0–15.7) | 0.003 |

CI, Confidence interval

^1^Southern European countries include Italy and Spain

^2^Northern, Western and Eastern European countries include Sweden, Romania, Belgium, France, Germany, Netherlands, Poland and United Kingdom

^3^Western style medicine includes general practitioners or family doctors, gastroenterologist, gynecologist, and surgeon.

^4^Traditional / folk healer includes folk healer or traditional healer, homeopathic doctor, ayurvedic doctor, traditional Chinese medicine doctor, and chiropractor.

All reported findings are based on univariate analyses.

**Supplementary table 4. Diet habits of the European population**

|  | Overall European population with at least one DGBI (n=8,170) | Southern European countries^1^ with at least one DGBI (n=1,818) | Northern, Western and Eastern European countries^2^ with at least one DGBI (n=6,352) | P-value |
| --- | --- | --- | --- | --- |
| **Food avoidance^3^, % (95% CI)** | 29.7 (26.7–32.6) | 29.7 (26.7–32.6) | NA | NA |
| **Food consumption (days/week)^4^, mean (95% CI)** | | | | |
| - - Milk products (milk, yogurt, or cheese) | 4.9 (4.8–4.9) | 4.9 (4.8–5.0) | 4.9 (4.8–4.9) | 0.190 |
| - - Meat from animals | 4.0 (3.9–4.0) | 3.3 (3.3–3.4) | 4.1 (4.1–4.2) | <0.001 |
| - - Fish | 1.5 (1.5–1.6) | 2.0 (1.9–2.1) | 1.4 (1.4–1.4) | <0.001 |
| - - Eggs | 2.2 (2.1–2.2) | 2.1 (2.0–2.1) | 2.2 (2.1–2.2) | 0.001 |
| - - Vegetables and legumes | 4.6 (4.6–4.7) | 4.3 (4.2–4.4) | 4.7 (4.7–4.8) | <0.001 |
| - - Fruits | 4.5 (4.4–4.5) | 4.8 (4.6–4.9) | 4.4 (4.3–4.4) | <0.001 |
| - - Bread (including pizza, pita and tortillas) | 5.2 (5.2–5.3) | 5.3 (5.2–5.4) | 5.2 (5.2–5.3) | 0.026 |
| - - Pasta | 2.6 (2.5–2.6) | 3.5 (3.4–3.6) | 2.3 (2.3–2.3) | <0.001 |
| - - Rice | 1.8 (1.8–1.8) | 2.0 (2.0–2.1) | 1.7 (1.7–1.8) | <0.001 |
| - - Tofu | 0.2 (0.2–0.3) | 0.2 (0.2–0.2) | 0.3 (0.2–0.3) | 0.044 |

CI, Confidence interval; NA, Not applicable

Southern European population includes the following countries: Italy and Spain

^1^Southern European countries include Italy and Spain

^2^Northern, Western and Eastern European countries include Sweden, Romania, Belgium, France, Germany, Netherlands, Poland and United Kingdom

^3^Food avoidance was reported exclusively by the Italian participants (n=2063) in response to the question: "In your experience, have you eliminated particular foods from your diet that you believe are responsible for your gastrointestinal disorders?"

^4^Food items consumption was assessed using the Food Frequency Questionnaire (FFQ)

All reported findings are based on univariate analyses.

**Supplementary Figure 1. Prevalence of DGBI across European countries**

**
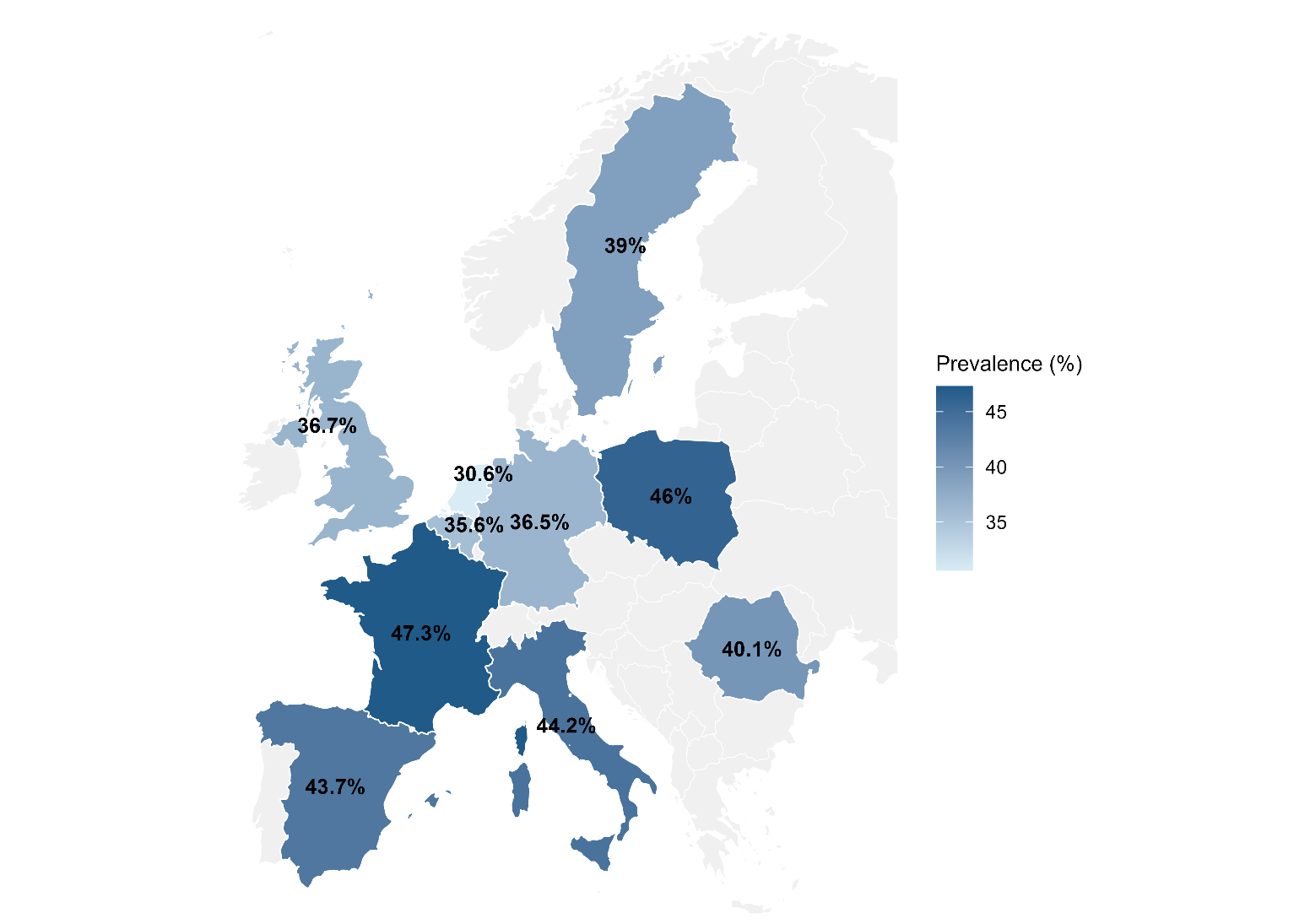
**

**Specific details of administered Questionnaires and classification used in the text**

Somatic symptoms were evaluated using a modified Patients Health Questionnaire-15 (PHQ-15), without three gastrointestinal-related items (i.e. stomach pain, constipation, loose bowels, or diarrhea, and nausea, gas or indigestion) and one menstrual symptom item, which was removed to ensure comparability between sexes. This questionnaire consists of a list of 11 common somatic symptoms, that constitute the majority of physical complaints in primary care. In addition, fibromyalgia was also assessed using a single question: "Have you ever been diagnosed by a doctor as having Fibromyalgia?".

The Patient-Reported Outcomes Measurement Information System (PROMIS) Global-10 was used to assess both physical and mental health-related quality of life, providing validated summary scores for each domain. Higher scores reflect better self-reported health status.

The Work Productivity and Activity Impairment: General Health (WPAI:GH) questionnaire consists of six questions, with a recall period of the previous seven days. Domain scores are expressed as percentages ranging from 0% to 100%, with higher values indicating greater impairment and reduced productivity. Items assessing work-related impairment (domains 1–3) were completed only by participants who were currently employed, whereas all respondents completed the activity impairment item. The WPAI:GH was administered in a subset of participating countries, namely Germany, the Netherlands, Italy, Poland, Spain, and Sweden. Inclusion of the WPAI:GH questionnaire was left to the discretion of the individual country principal investigators. Consequently, all analyses involving work productivity and activity impairment outcomes were restricted to data derived from these countries.

Psychological distress was evaluated using the Patients Health Questionnaire-4 (PHQ-4), a validated tool designed to measure symptoms of anxiety and depression over the past two weeks. The PHQ-4 consists of four questions scored on a four-point Likert scale, ranging from 0 ("not at all") to 3 ("nearly every day"). The total score, ranging from 0 to 12, reflects levels of anxiety, depression, and overall psychological distress, with higher scores indicating greater severity of distress. We also used a dichotomous variable to distinguish between participants with any level of psychological distress [mild (score: 3-5), moderate (score: 6-8), or severe (score: 9-12)] and those with a normal status (score: 0-2) according to validated cutoff points.

Concern and embarrassment regarding bowel functioning were assessed using the following two questions: "Are you concerned about your bowel functioning?", and "Are you embarrassed to discuss your bowel functioning with others?".

Healthcare utilization was further assessed by capturing the self-reported frequency of physician visits, both in general and specifically for gastrointestinal complaints. In addition, participants were asked to report their use of medications for symptoms commonly associated with gastrointestinal disorders, including constipation, diarrhea, nausea, gastric acidity, bloating, and excessive gas. To gain deeper insight into different patterns of healthcare-seeking behaviors, participants were also asked to report the frequency with which they used Western-style medicine, which included general practitioners or family doctors, gastroenterologists, gynecologists, and surgeons; and traditional or folk medicine, which encompassed visits to folk or traditional healers, homeopathic doctors, ayurvedic doctors, traditional Chinese medicine practitioners, and chiropractors.

A Food Frequency Questionnaire (FFQ) was utilized to evaluate the weekly consumption frequency of various food groups, including milk products, animal-based meats, fish, eggs, vegetables and legumes, fruits, bread (including pizza, pita, and tortillas), pasta, rice, and tofu. Participants reported the number of days per week (0–7) they consumed each food group.

Residential area classification was based on two separate items: (1) self-reported living environment (urban vs. rural), and (2) the size of the local community, categorized as city (>50,000 inhabitants), town (2,500–50,000 inhabitants), and village/small town (<2,500 inhabitants).
